# EPR Studies of Aβ42 Oligomers Indicate a Parallel In-Register β-Sheet Structure

**DOI:** 10.1021/acschemneuro.3c00364

**Published:** 2023-12-18

**Authors:** Chelsea Jang, Diana Portugal Barron, Lan Duo, Christine Ma, Hanna Seabaugh, Zhefeng Guo

**Affiliations:** Department of Neurology, Brain Research Institute, David Geffen School of Medicine, University of California, Los Angeles, Los Angeles, California 90095, United States

**Keywords:** alzheimer’s disease, protein aggregation, electron paramagnetic resonance, amyloid, ADDLs, oligomers

## Abstract

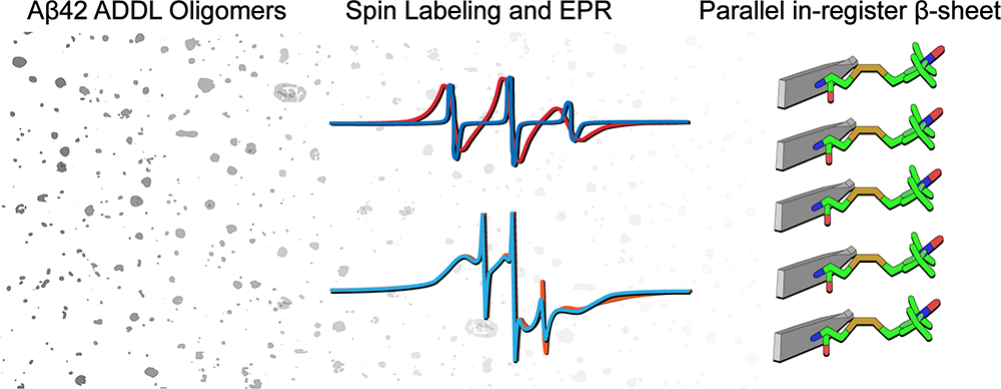

Aβ aggregation leads to the formation of both insoluble amyloid fibrils and soluble oligomers. Understanding the structures of Aβ oligomers is important for delineating the mechanism of Aβ aggregation and developing effective therapeutics. Here, we use site-directed spin labeling and electron paramagnetic resonance (EPR) spectroscopy to study Aβ42 oligomers prepared by using the protocol of Aβ-derived diffusible ligands. We obtained the EPR spectra of 37 Aβ42 oligomer samples, each spin-labeled at a unique residue position of the Aβ42 sequence. Analysis of the disordered EPR components shows that the N-terminal region has a lower local structural stability. Spin label mobility analysis reveals three structured segments at residues 9–11, 15–22, and 30–40. Intermolecular spin–spin interactions indicate a parallel in-register β-sheet structure, with residues 34–38 forming the structural core. Residues 16–21 also adopt the parallel in-register β-structure, albeit with weaker intermolecular packing. Our results suggest that there is a structural class of Aβ oligomers that adopt fibril-like conformations.

## Introduction

Deposition of Aβ fibrils in the form of amyloid plaques is a pathological hallmark of Alzheimer’s disease.^1,2^ In addition to insoluble fibrils,^3^ Aβ aggregation, a supersaturation-driven process,^4^ also leads to the formation of soluble aggregates, collectively referred to as oligomers.^5−7^ Biochemical and biological assays suggest that Aβ oligomers play a crucial role in the pathogenesis of Alzheimer’s disease.^8−11^ Soluble Aβ oligomers are better correlated with disease progression than amyloid fibrils.^12^ Targeting these oligomers, therefore, may prove to be an effective therapeutic strategy for the prevention and treatment of Alzheimer’s disease.

Aβ protein is the product of proteolytic cleavage of the amyloid precursor protein by β- and γ-secretases.^13^ Due to the mechanism of sequential digestion by γ-secretase at the C-terminal end of Aβ protein,^14^ multiple Aβ isoforms are produced with different C-terminal residues. The 40-residue Aβ40 and 42-residue Aβ42 are the two main Aβ isoforms. The only difference between Aβ40 and Aβ42 is that Aβ42 has two more residues at the C-terminal end. Despite the total concentration of Aβ40 being several fold higher than Aβ42,^15,16^ Aβ42 is the major Aβ species in the parenchymal plaques.^17,18^ Conversely, the major Aβ species in the cerebrovascular plaques is Aβ40.^17,18^ The concentration ratio of Aβ42 to Aβ40 has been suggested to be a better indicator than the absolute Aβ42 levels in the diagnosis of Alzheimer’s disease.^19^

To characterize the structure and physicochemical properties of Aβ oligomers and study their biological activities, different protocols have been developed to prepare relatively stable oligomers that do not readily convert to amyloid fibrils. Aβ-derived diffusible ligands (ADDLs)^20^ are among the most commonly used Aβ oligomers in the studies of animal and cellular models of Alzheimer’s disease.^21−25^ Aβ42 oligomers have also been prepared in the presence of detergents, such as sodium dodecyl sulfate (SDS), which is used to make globulomers,^26^ and dodecyl phosphocholine (DPC) used to form β-barrel pore-forming Aβ oligomers.^27^ ADDL preparation has advantages over detergent-based oligomers because detergents have been found to affect native protein structure and stability.^28^ It is worth noting that all of the in vitro oligomer preparations may have inadvertently led to the formation of oligomers that are structurally and functionally distinct from the in vivo transient oligomers. Because the in vivo oligomers are inaccessible to direct structural characterization, conformation-sensitive antibodies have been used as a main approach to establish the structural similarity between in vitro and in vivo oligomers. For example, ADDL-specific antibodies have been shown to detect brain-derived oligomers.^29^ However, until highly sensitive tools are developed to characterize the transient aggregation species in vivo, questions will remain as to how the in vitro aggregation procedures may affect the intrinsic aggregation behavior of the Aβ protein.

Various biochemical and biophysical studies have been performed to characterize ADDLs, but key details of their molecular structure remain unclear or uncharacterized. Fourier transform infrared spectroscopy (FTIR) studies^30^ reported spectral signatures of antiparallel β-sheet structures in ADDLs, but did not identify specific regions for these structures. A hydrogen exchange study^31^ of ADDLs showed that residues 15–24 and 29–42 have 50–70% protection, while residues 25–28 are unprotected, suggesting a β-turn-β structure for residues 15–42. Studies using atomic force microscopy (AFM), size exclusion chromatography (SEC), and light scattering suggest a very broad range of ADDL sizes ranging from only a few Aβ subunits to hundreds.^32−36^ The lack of protocol standardization and high variability in ADDL preparations make it difficult to draw conclusions across different studies and limit the structural understanding.

**Figure 1:**
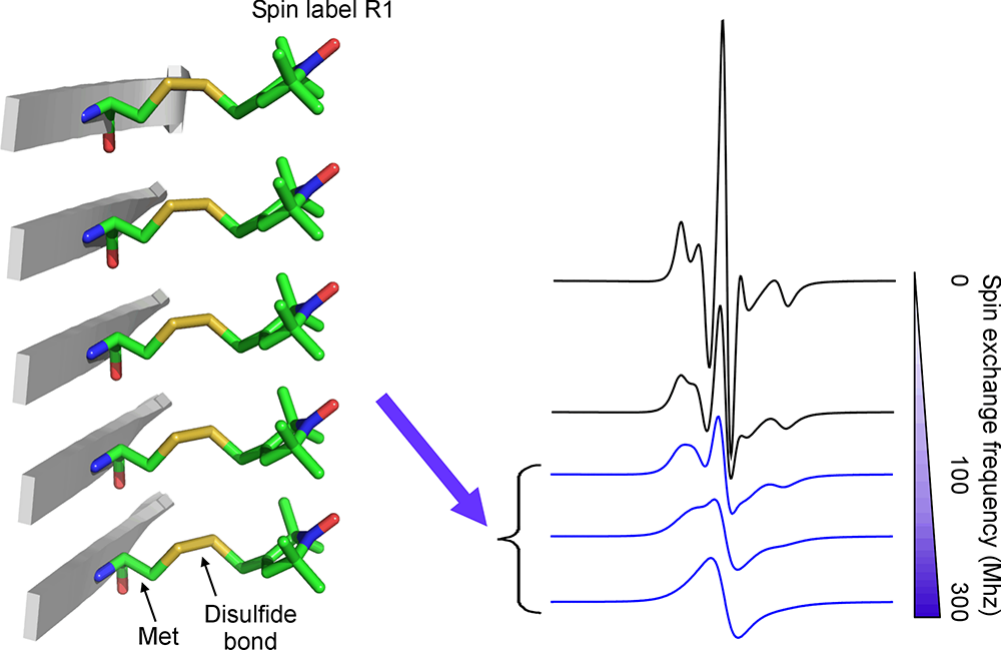
EPR spectral features in a parallel in-register β-sheet. Left, spin label R1 is modeled on a parallel β-sheet structure. Right, simulated EPR spectra with varying strength of spin–spin interactions (expressed in spin exchange frequency). Note that spin labels in a parallel in-register β-sheet structure leads to strong spin–spin interactions with spin exchange frequencies of 100 MHz or higher, which is characterized by a single-line EPR spectral feature (blue spectra).

In this work, we employed site-directed spin labeling and electron paramagnetic resonance (EPR) spectroscopy to characterize Aβ42 oligomers prepared with the ADDL protocol. This process begins by introducing a cysteine residue at the position of interest through site-directed mutagenesis, which is then covalently modified with a spin labeling reagent. The EPR spectrum produced by the spin-labeled Aβ reports structural and dynamic features of the local environment at the labeling site. In amyloid fibrils, the intermolecular spin–spin interaction in the parallel in-register β-sheet gives rise to a characteristic single-line EPR spectral feature, as illustrated in Figure 1. In the absence of spin–spin interactions, the EPR spectrum of R1, a commonly used spin label, has three resonance lines (Figure 1, black spectra). With increasing spin–spin interaction, the three lines collapsed toward the center line to become a single-line spectrum (Figure 1, blue spectra). The strength of the spin–spin interaction is quantified using spin exchange frequency, a parameter that can be extracted by simulating the experimental EPR spectra. To comprehensively study the structure of Aβ42 ADDL oligomers, we spin-labeled 37 unique positions along the length of the Aβ42 sequence with the R1 spin label. The results indicate that the Aβ42 ADDL oligomers adopt a parallel in-register β-sheet structure over the entire Aβ42 sequence. Residues 34–38 show the strongest interstrand spin–spin interactions, suggesting a tightly packed structural core at these residues. Similarly, residues 16–21 form a second well-packed β-sheet, while the rest of the sequence appears to adopt less ordered structures.

## Results and Discussion

### Site-Directed Spin Labeling of Aβ42 ADDL Oligomers

Using site-directed spin labeling, we obtained 42 spin-labeled Aβ42 variants covering every residue position of the Aβ42 sequence. We then prepared oligomers using the standard ADDL protocol (see Methods section).^32−34^ Out of the 42 preparations, 37 variants yielded sufficient amounts of oligomers for subsequent structural studies.

**Figure 2:**
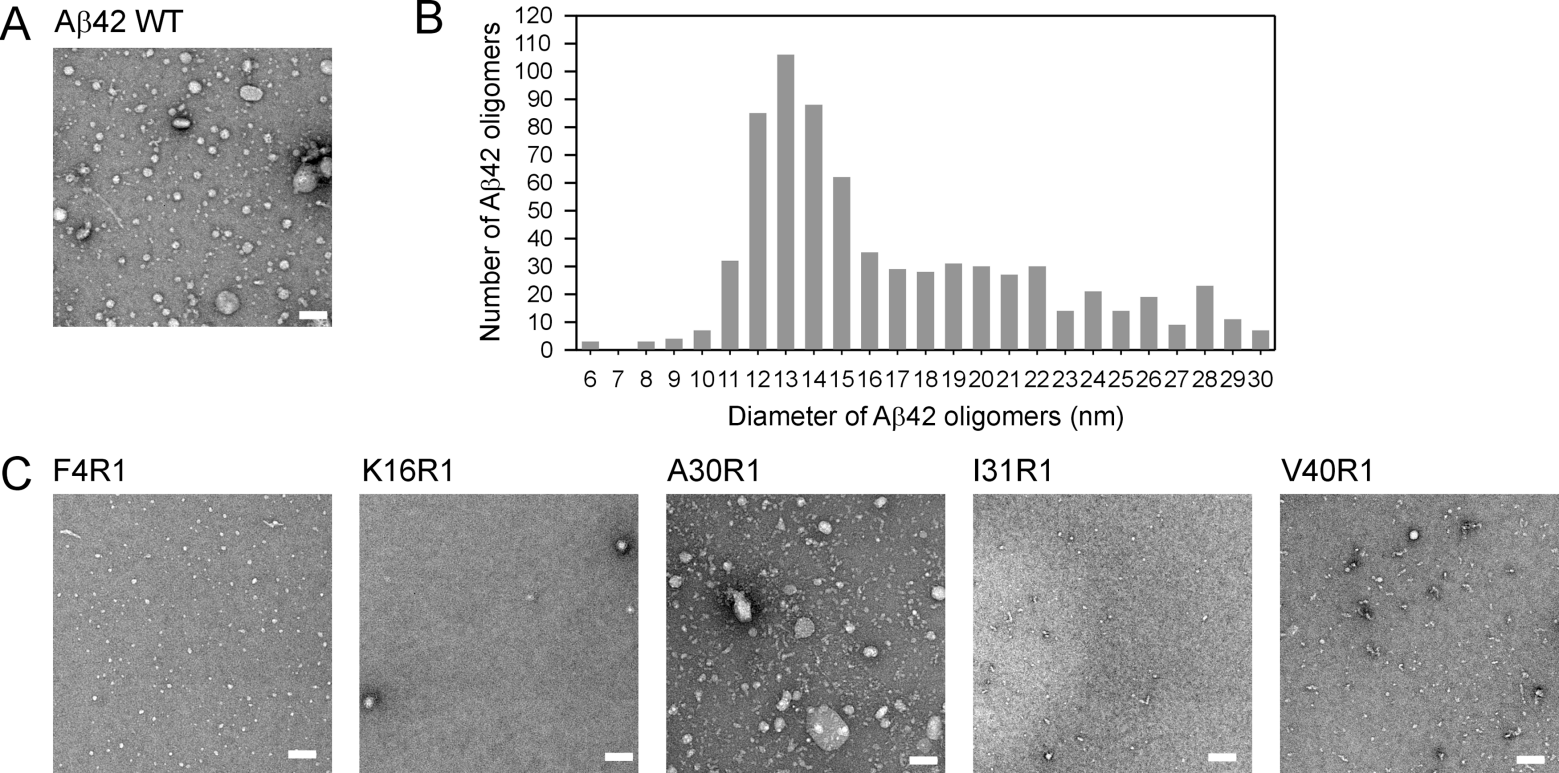
Transmission electron microscopy studies of wild-type and spin-labeled Aβ42 ADDL oligomers. (A) TEM studies of wild-type Aβ42 ADDL oligomers. (B) Size distribution of wild-type Aβ42 ADDL oligomers. (C) TEM studies of spin-labeled Aβ42 ADDL oligomers. R1 represents the spin label.

We performed transmission electron microscopy (TEM) to characterize the morphology of the Aβ42 oligomers. As shown in Figure 2A, wild-type Aβ42 oligomers show predominantly globular structures with diameters ranging from 6 to 30 nm. The majority of the Aβ42 oligomers observed had diameters of 12–15 nm (Figure 2B). For spin-labeled Aβ42 oligomers (Figure 2C), the size varied between the different spin-labeled mutants. The overall morphology of both wild-type and spin-labeled Aβ42 oligomers was globular, with a small proportion of curvilinear structures. These results suggest that spin labeling in general does not disrupt Aβ42 oligomer formation, although TEM studies do not reveal how spin labeling affected the detailed molecular structure. These results are consistent with previous spin labeling studies,^37−44^ where spin-labeled Aβ generally behaved like wild-type Aβ in forming oligomers and fibrils. Previous studies also showed that spin labeling at certain residue positions affected the kinetics of aggregation,^38^ but whole data analysis revealed that the overall structure was not disrupted by spin labeling.^37−45^

A previous AFM study by Mustata et al.^36^ showed that ADDLs have diameters at approximately 3 nm at 10 μM Aβ42 concentrations. A dynamic light scattering study by Limbocker et al.^25^ showed an average diameter of approximately 22 nm for ADDLs, ranging from 15 to 50 nm. Another dynamic light scattering study by Hepler et al.^46^ showed a 150 to 1000 kDa size range for ADDLs. The overall findings from these studies suggest that Aβ42 ADDLs have a broad range of size distributions. In addition, size distribution of ADDLs has been shown to be Aβ concentration-dependent.^36,47^ The ADDL oligomers prepared in this work are consistent with those in previous studies.

**Figure 3:**
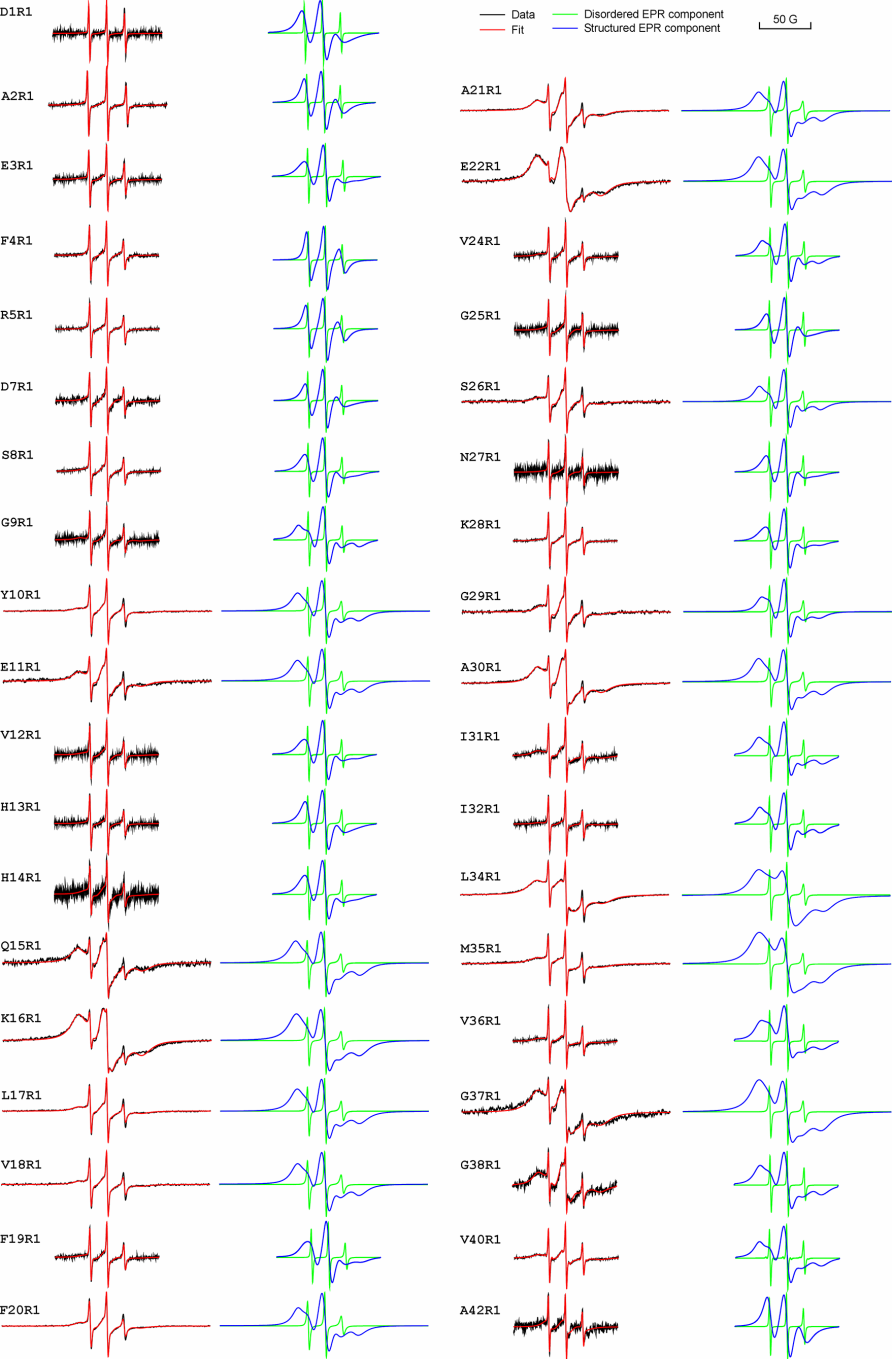
EPR spectra of spin-labeled Aβ42 ADDL oligomers. Experimental spectra (black traces) are overlaid on the best fits from spectral simulations (red traces). Each EPR spectrum consists of two spectral components, corresponding to a disordered state (green) and a structured state (blue). All spectra are scaled to the center line amplitude. The scan width is either 100 or 200 G, optimized for each individual sample.

EPR spectra were collected on 37 spin-labeled Aβ42 ADDL oligomer samples. The EPR data are shown in Figure 3 (black traces). We analyzed the EPR spectra by performing spectral simulations to obtain the best fits to the experimental spectra (Figure 3, red traces). Spectral simulations reveal two components for all 37 samples corresponding to two structural states of the spin label. One EPR spectral component has three sharp lines, indicative of a spin label with rapid motion, which corresponds to disordered protein structures (Figure 3, green traces). Another component has broad spectral lines corresponding to an ordered structure (Figure 3, blue traces). Spectral simulations also allow us to extract the relative abundance of the disordered and structured components, as well as the strength of spin–spin interactions in the oligomers. These extracted parameters are further discussed below.

**Figure 4:**
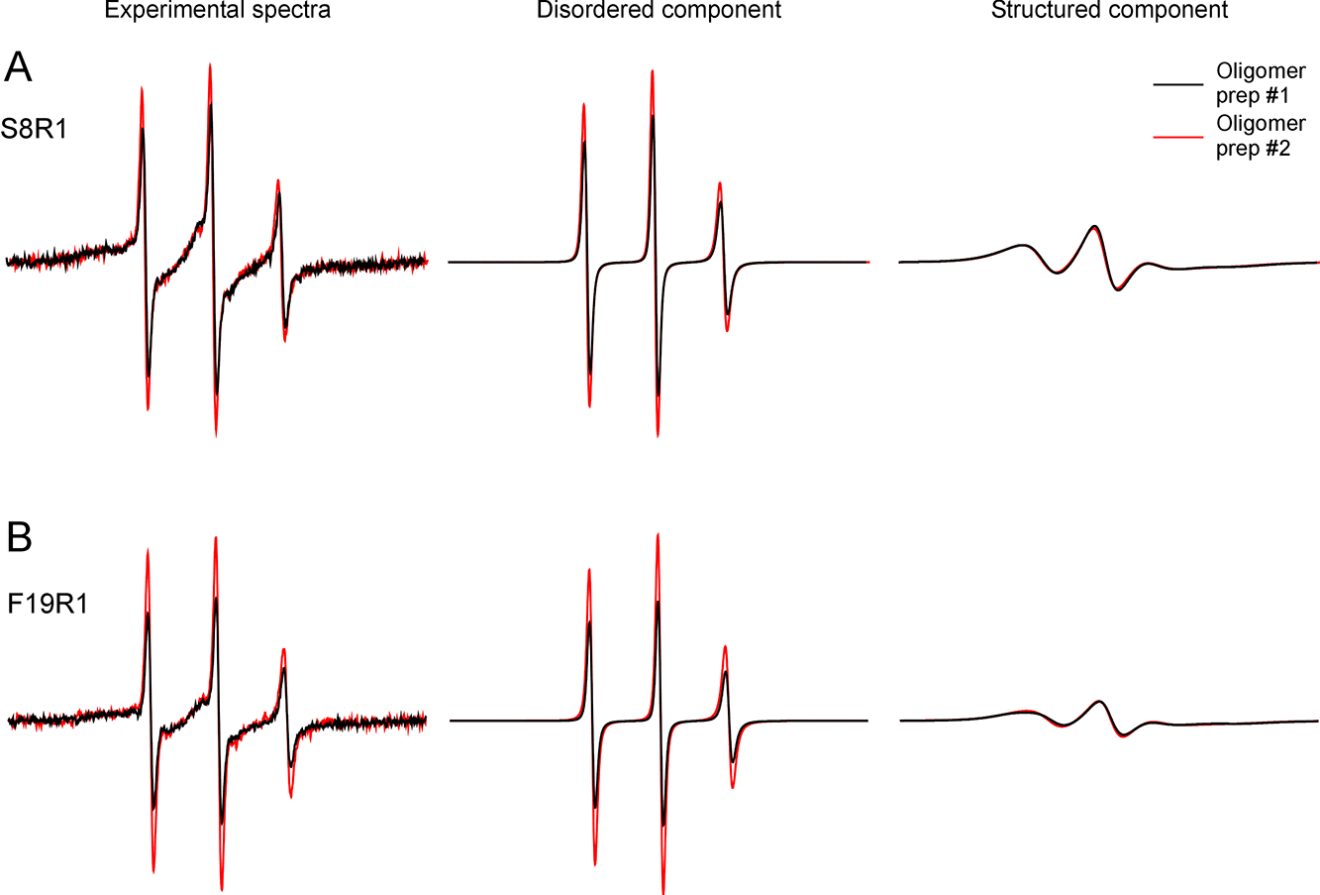
Comparison of the EPR spectra from two preparations of Aβ42 ADDL oligomers. (A) Aβ42 oligomers spin-labeled at residue 8. (B) Aβ42 oligomers spin-labeled at residue 19. Note that the structured spectral components from two oligomer batches are superimposable, while the different spectral amplitude of the three sharp lines suggests different amounts of the disordered component.

As a control experiment, we investigated the reproducibility of the EPR data for spin-labeled Aβ42 ADDL oligomers. We prepared ADDL oligomers twice using the same protocol on separate days. We studied two spin-labeled Aβ42 mutants, S8R1 and F19R1, and found that the only difference between the two oligomer preparations was the abundance of the disordered component (Figure 4). The EPR line shape of the structured component remained unchanged from one preparation to the next (Figure 4). The high reproducibility of the structured EPR component suggests that even though the ADDL oligomers have a wide range of sizes, they adopt specific structures.

**Figure 5:**
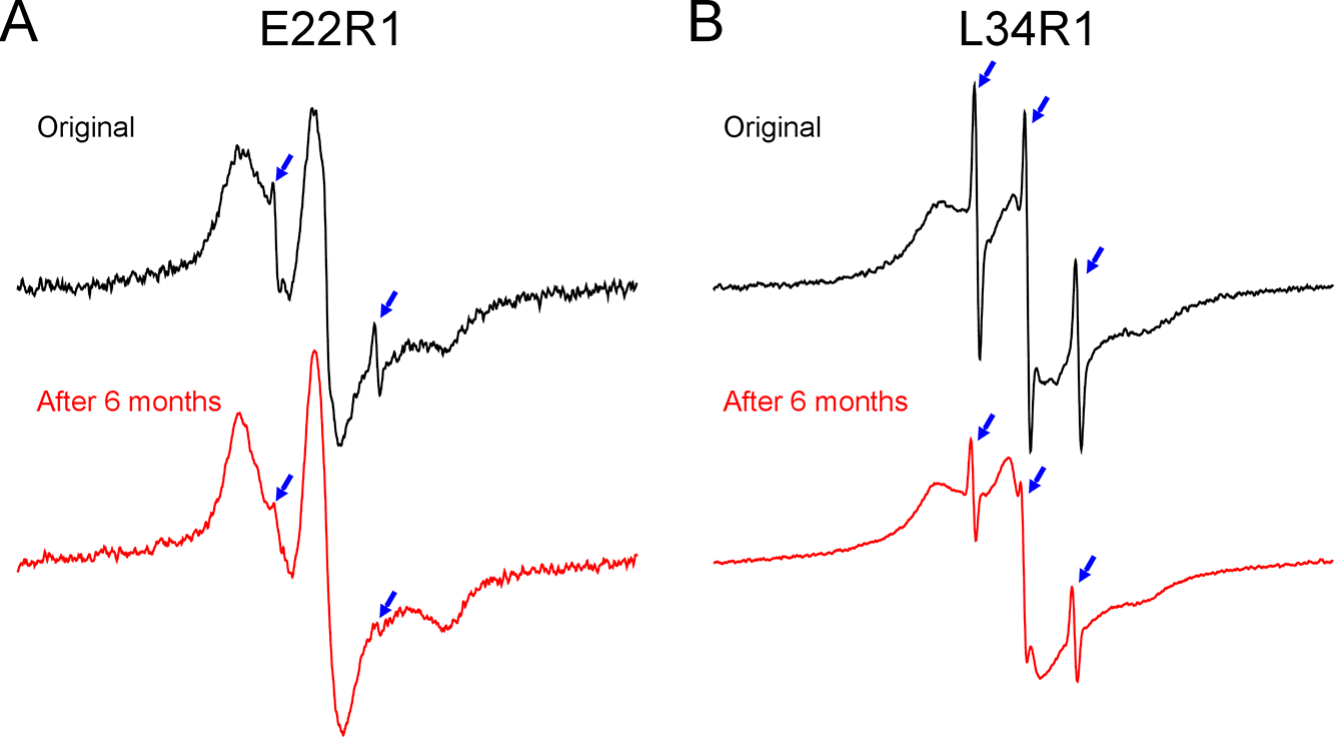
Assessment of the structural stability of Aβ42 ADDL oligomers. EPR spectra of Aβ42 E22R1 (A) and L34R1 (B) before and after incubation at 4 °C for more than 6 months. Note that the only notable difference in the after-incubation spectra is a slight reduction of the disordered component (indicated by blue arrows), while the overall line shape remains unchanged, suggesting a stable structure for Aβ42 ADDLs.

To assess the structural stability of spin-labeled Aβ42 ADDLs, we performed EPR studies on some selected ADDL samples after more than 6 months of incubation at 4 °C in the EPR capillary tubes. Two representative samples, Aβ42 E22R1 and L34R1, show that overall EPR spectra remain unchanged before and after incubation, suggesting highly stable structures for ADDLs at 4 °C (Figure 5). The only notable difference is that incubation led to a slight reduction in the disordered component, suggesting some structural ordering over time. Our work is consistent with previous studies, which noted that ADDLs remain oligomeric even with incubation at 37 °C for 24 h.^34^

### Local Structural Stability in Aβ42 Oligomers

**Figure 6:**
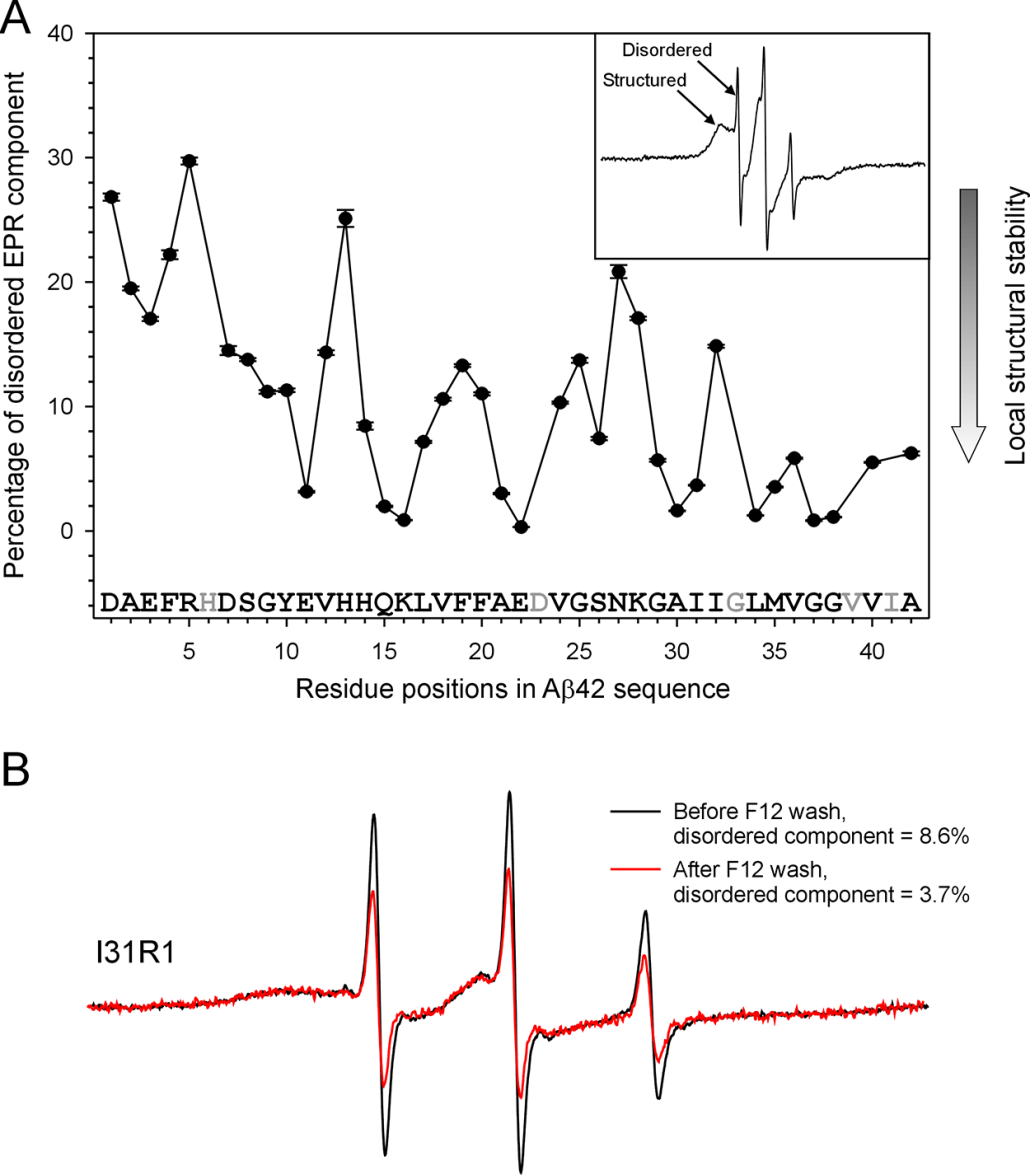
The disordered EPR component represents local structural stability in Aβ42 ADDL oligomers. (A) Plot of the percentage of the disordered component from spectral simulations as a function of residue positions and (B) EPR spectra of Aβ42 oligomers before and after wash with F12 medium.

The percentage of the disordered component obtained from spectral simulations is plotted as a function of Aβ42 residue positions in Figure 6A. This percentage represents the proportion of total labeled Aβ42 molecules in the solution that adopt disordered confirmations. There are two main factors contributing to the disordered EPR spectral component: the presence of Aβ42 monomers and the local unfolding at the labeling site. Figure 6A shows that the percentage of the disordered component is highly dependent on the residue position. There is a general trend of a higher proportion of disordered components at the N-terminal region of Aβ42, while the central and C-terminal regions show a smaller proportion of disordered components. The influence of dissociated monomers on the structural composition of the oligomer is expected to be uniform across different residue positions. Therefore, this pattern suggests that the disordered components are significantly influenced by local structural disorder.

To evaluate the contribution of monomers to the disordered EPR component, we washed an oligomer sample with an F12 medium. Immediately after the wash, we performed EPR measurements and observed that the reduction of monomers decreased but did not eliminate the amount of the disordered component (Figure 6B). This is consistent with the notion that the disordered component reflects the local structural stability.

Most labeling sites before residue 14 have 10% or more disordered EPR components, suggesting that the N-terminal region has low local structural stability (Figure 6A). In contrast, a majority of residues after 14 have higher local stability with less than 10% disordered components. It is worth noting that within the C-terminal region, residues 24–28 have a higher percentage of disordered components. The EPR results are consistent with a previous hydrogen exchange study of ADDLs by Pan et al.,^31^ which showed that the N-terminal residues 2–14 are weakly hydrogen-bonded, while the C-terminal residues 15–42, excluding residues 25–28, display strong backbone hydrogen bonding.

### Structured Segments in Aβ42 Oligomers

**Figure 7:**
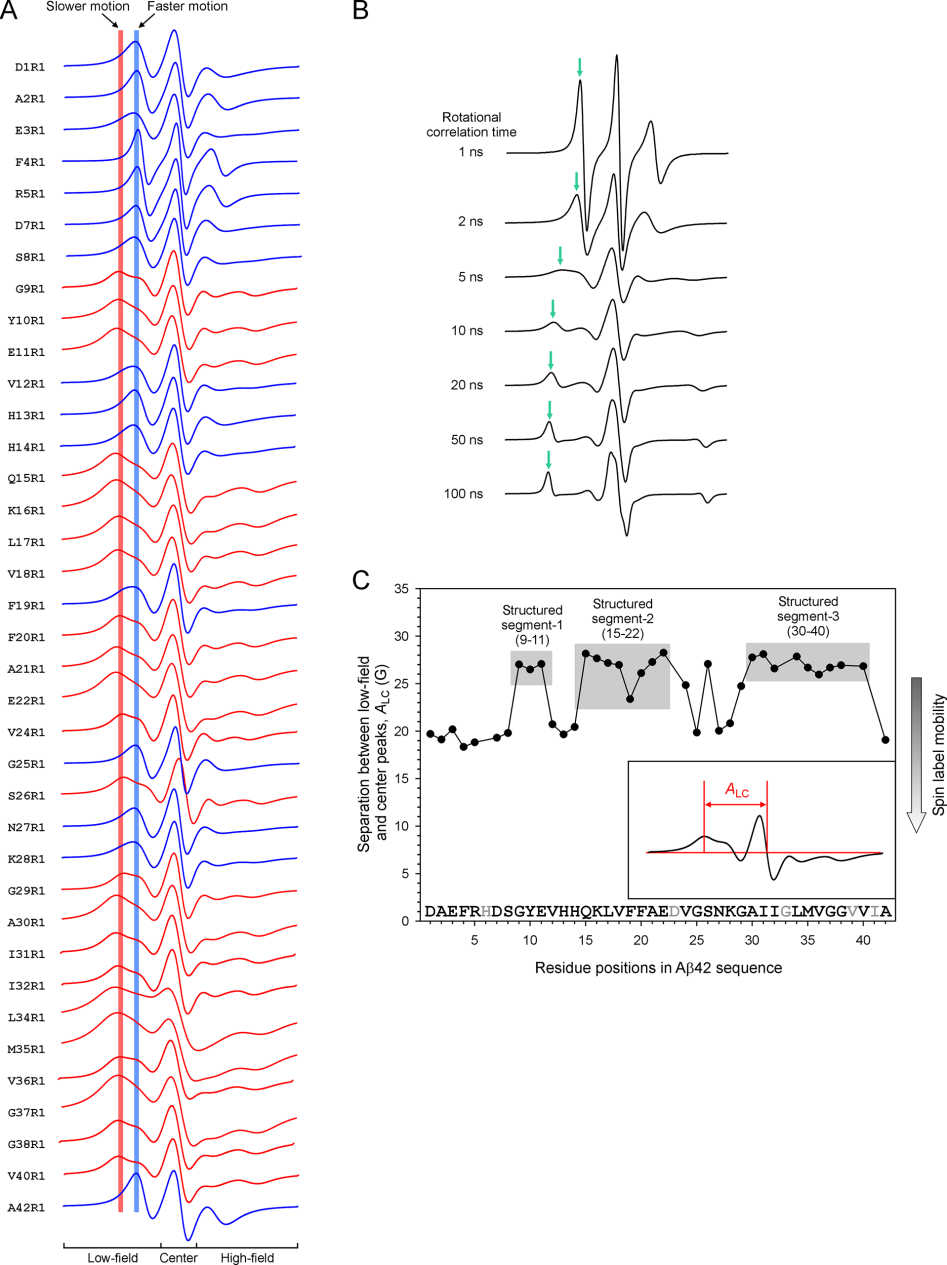
Lineshape comparison of the structured EPR spectral component reveals three structured segments in Aβ42 ADDL oligomers. (A) EPR spectra of the structured component from spectral simulations. These spectra can be categorized to have either slow (red vertical line) or fast motion (blue vertical line) based on the position of their low-field peaks. The EPR spectra are colored based on their low-field peak positions. (B) Simulated EPR spectra with spin label mobility ranging from rotation correlation time of 1 to 100 ns. (C) Plot of the separation between low-field peak and the center peak, called *A*_CL_, as a function of residue positions.

We graphed together all of the structured EPR spectral components from the simulated EPR spectra in Figure 7A. Qualitatively, these EPR spectra can be separated into two groups based on the position of their low-field peak. Spin labels with faster motion give rise to EPR spectra with low-field peaks closer to the center line (Figure 7A, blue vertical line), while spin labels with slower motion show low-field peaks farther from the center line (Figure 7A, red vertical line). Using this simple metric, we can separate these spectra into two categories: slower motion (Figure 7A, red spectra) and faster motion (Figure 7A, blue spectra). To help visualize the effect of spin label motion on the EPR spectral line shape, a series of simulated EPR spectra with defined rotational correlation times are shown in Figure 7B.

To quantitatively analyze these EPR spectra, we plotted the separation between the low-field and center peaks as a function of residue positions in Aβ42 (Figure 7C). The separation between the low-field and center peaks is a function of spin label mobility, with low spin label mobility (high structural order) corresponding to a larger separation of low-field and center peaks. From this plot, we identified three structured segments: segment-1 (residues 9–11), segment-2 (residues 15–22), and segment-3 (residues 30–40). Structured segments 2 and 3, separated by a disordered region (residues 24–29), resemble the β-turn-β structure commonly observed in Aβ42 fibrils. The EPR data suggest that, at least at the secondary structure level, residues 15 to 42 in Aβ42 ADDL oligomers have structural features similar to those of Aβ42 fibrils.

### Intermolecular Spin–Spin Interactions Suggest a Parallel In-Register β-Sheet Structure

The EPR spectra of Aβ42 ADDLs at several residue positions reveal strong intermolecular spin exchange interactions, which are characterized by the upward shift of the low-field peak and the simultaneous downward shift of the high-field peak (Figure 1). We have previously shown that spin-labeled Aβ42 fibrils show characteristic single-line EPR spectra due to the strong spin exchange interactions in the parallel in-register β-sheet structure.^37−41^ Single-line EPR spectra have been widely used as a diagnostic feature for the parallel in-register β-sheet structure of amyloid fibrils.^48^

**Figure 8:**
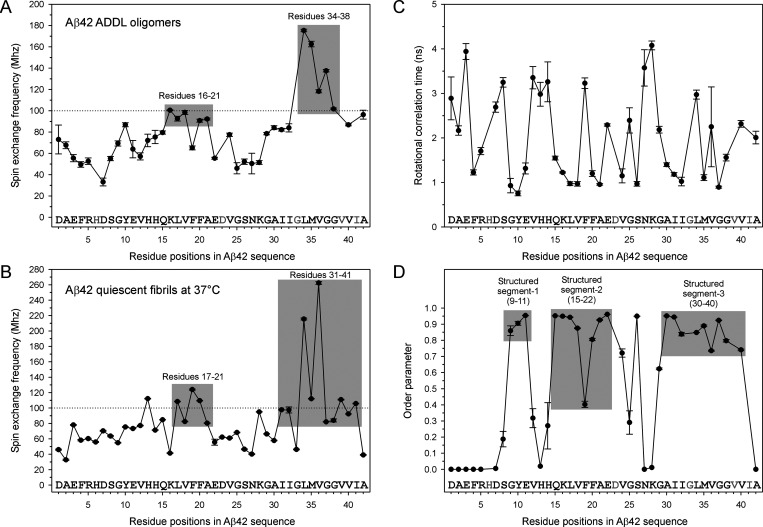
Intermolecular spin–spin interactions in Aβ42 ADDL oligomers. (A) Plot of residue-specific spin exchange frequency in Aβ42 ADDL oligomers. (B) Plot of residue-specific spin exchange frequency in Aβ42 fibrils. (C) Plot of rotational correlation time in Aβ42 ADDL oligomers. (D) Plot of residue-level order parameter in Aβ42 ADDL oligomers.

At several residue positions of the Aβ42 oligomers (e.g., L34R1, M35R1, and G37R1), the EPR spectra display single-line characteristics similar to those of fibrils. To quantitatively analyze the intermolecular spin–spin interactions, we extracted the spin exchange frequencies at each residue using spectral simulations. Generally, we consider an exchange frequency of 100 MHz or higher as indicative of strong spin exchange interactions. The residue-specific exchange frequency plot (Figure 8A) of Aβ42 ADDLs had strong spin exchange interactions at residues 34–38, with frequencies between 100 and 180 MHz. Similarly, residues 16–21 displayed frequencies near 100 MHz, indicating another section of relatively strong exchange interactions. For comparison, we plotted the residue-specific spin exchange frequencies in Aβ42 fibrils prepared at 37 °C without agitation using previously published data^39^ (Figure 8B). In fibrils, the region with the strongest intermolecular spin exchange interactions is composed of residues 31–41. Residues 17–21 form a second region with strong exchange interactions. Although the oligomers show a similar profile of residue-specific exchange frequency to fibrils, they possess weaker interactions across the entire sequence.

The similarity in characteristic single-line EPR spectra, invariably observed in spin-labeled amyloid fibrils with parallel in-register β-sheet structures,^37,40,49^ suggest that Aβ42 ADDL oligomers also adopt parallel in-register β-sheet structures. For both oligomers and fibrils, the C-terminal hydrophobic region appears to have the strongest interstrand packing within the parallel in-register β-sheet structure. Therefore, from the perspective of intermolecular side-chain packing, the oligomers and fibrils adopt highly similar structures.

The plot of the rotational correlation time as a function of residue position in Aβ42 ADDL oligomers does not reveal any particular trends (Figure 8C). Interestingly, the order parameter plot (Figure 8D) in ADDLs shows three highly ordered regions that resemble those in Figure 7C. In this work, the spin label motion is simulated using a “wobbling in a cone” model and the order parameter describes the size of the cone.^50^ The trends in order parameters suggest that the structured regions in ADDLs are characterized by high-frequency, low-amplitude motions, in contrast to low correlation time.

### Comparison with Structures of Other Oligomers and Fibrils

**Figure 9:**
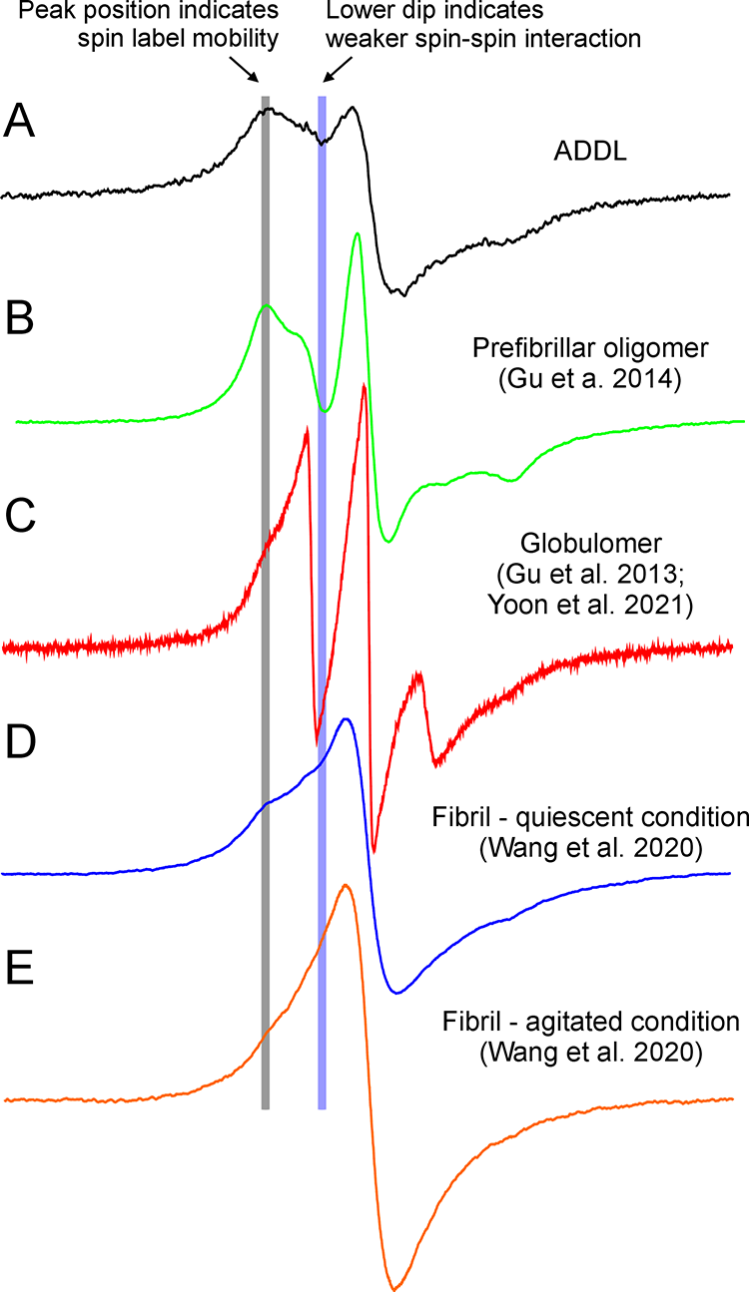
EPR spectra of Aβ42 spin-labeled at position 34 in various types of aggregates. (A) Aβ42 ADDL oligomers (this work). (B) Aβ42 prefibrillar oligomers (Gu et al.^1^). (C) Aβ42 globulomers (Gu et al.^2^ and Yoon et al.^3^). (D) Aβ42 quiescent fibrils (Wang et al.^4^). (E) Aβ42 agitated fibrils (Wang et al.^4^). Vertical lines are drawn to show the spectral features that distinguish different types of Aβ42 aggregates. The gray vertical line indicates the position of the outermost EPR peak, which correlates with spin label mobility. The blue vertical line indicates the dip between the lower-field and center-field lines. Stronger intermolecular spin–spin interactions lead to shallower dips.

We have previously used spin labeling and EPR to study the Aβ42 oligomers prepared using the protocols of prefibrillar oligomers^45^ and globulomers.^43,44^Figure 9 shows a comparison of the EPR spectra of the three oligomer types: ADDLs, prefibrillar oligomers, and globulomers. Prefibrillar oligomers, which bind to the A11 antibody,^51^ are immunologically distinctive from fibrillar oligomers, which bind to the OC antibody.^52^ The A11 antibody recognizes a generic epitope that is present on the oligomers of different amyloid proteins including Aβ, α-synuclein, and insulin and is not reactive to monomers or fibrils.^51^ On the other hand, the OC antibody recognizes A11-negative fibrillar oligomers and fibrils.^52^ The ADDLs (Figure 9A) show spin label mobility similar to that of prefibrillar oligomers (Figure 9B), characterized by the position of the low-field peak (Figure 9B, indicated by the gray vertical line). Another characteristic of ADDLs is that the low-field and high-field EPR peaks collapse toward the center line, forming a single-line EPR spectrum, suggesting strong intermolecular spin–spin interactions (Figure 9A, indicated by the purple vertical line). In contrast, the prefibrillar oligomers show a well-separated three-line EPR spectrum (Figure 9B). Aβ42 globulomers are a type of oligomer prepared in the presence of low concentrations of SDS.^26^ The globulomers show an EPR spectrum with both faster motion and weaker spin–spin interactions than those of ADDLs (Figure 9C). Our previous studies revealed that globulomers lack a well-packed structural core based on the overall faster motion at all labeling sites.^44^ Previous EPR studies suggest that both prefibrillar oligomers and globulomers adopt antiparallel β-sheet structures.^43−45^ The EPR data of ADDLs in this work, in contrast to other oligomers, suggest that ADDLs adopt a parallel in-register β-sheet structure.

In the amyloid fibrils, the EPR spectra are characterized by their single-line feature (Figure 9D,E). Instead of three well-separated peaks, the EPR spectra show a single peak at the center-field position. Quiescent Aβ42 fibrils show a small bump at the low-field position (Figure 9D, indicated by the gray vertical line), while agitated fibrils lack this feature, showing a completely smoothed-out single-line spectrum (Figure 9E). This suggests that agitated fibrils have an overall more compact intermolecular packing than quiescent fibrils. In comparison, the ADDL EPR spectrum shows a larger bump at the low-field position (Figure 9A), suggesting a more loosely packed β-sheet in the oligomers than fibrils.

Previously, Pan et al.^31^ performed a hydrogen exchange mass spectrometry study of Aβ42 ADDLs. They found that amide hydrogens at residues 15–24 and 29–42 have 50–70% protection, suggesting the formation of strong hydrogen bonds along the backbones of these residues. Residues 25–28 are completely unprotected from hydrogen exchange. The hydrogen exchange data are consistent with a β-turn-β structure for residues 15–42, with residues 25–28 forming the turn between two β-strands at 15–24 and 29–42. The residue-specific hydrogen protection profile closely matches spin label mobility analysis in Figure 7, which shows two structured segments at residues 15–22 and 30–40. We found that N-terminal residues 1–14 have substantial intermolecular spin–spin interactions, consistent with the findings by Pan et al.^31^ that the N-terminal residues are partially protected from hydrogen exchange.

Studies of Aβ42 oligomers that are prepared with ADDL-like protocols suggest that parallel β-sheets may be a consensus structural feature for these oligomers. Parthasarathy et al.^53^ used solid-state NMR to study a type of Aβ42 oligomer called amylospheroid. They used a preparation protocol that is very similar to the ADDL protocol. In a typical ADDL protocol,^32−34^ Aβ42 is first dissolved in DMSO, and then diluted to phenol red-free F12 medium, followed by incubation at 4 °C for 24 h without agitation. The protocol in Parthasarathy et al.^53^ varies from the typical ADDL protocol by doing a 14 h incubation at 4 °C with slow rotation following the DMSO to F12 dilution. Solid-state NMR data^53^ suggest that Aβ42 adopts a parallel β-sheet structure in these oligomers, although the intermolecular distance measurements suggest that the parallel β-sheets may not be in-register. Using a slightly different preparation protocol, Xiao et al.^54^ prepared spherical oligomers by diluting the DMSO-solubilized Aβ42 to a low-salt buffer (10 mM phosphate, pH 7.5) and incubating the sample at 4 °C for 12–14 h with 400-rpm circular agitation. It is worth noting that ADDLs can also be prepared by diluting an Aβ42 stock solution in DMSO to a PBS buffer.^55,56^ Solid-state NMR data^54^ suggest that these Aβ42 spherical oligomers also adopt parallel β-sheet structures with likely off-register arrangements, judging from weaker intermolecular interactions. The EPR studies in this work show weaker intermolecular spin–spin interactions in the Aβ42 ADDL oligomers than in fibrils, which we attribute to the more loosely packed structures of the oligomer. Even in fibrils, the EPR spectra at different residue positions show spin exchange interactions of variable strength, even though they all adopt parallel in-register β-sheet structures. As a result, we conclude that Aβ42 adopts parallel in-register β-sheet structures in ADDL oligomers.

Previously, Kayed et al.^52^ studied the binding of ADDLs to the OC antibody, which recognize fibrils and fibrillar oligomers. They found that SEC fractions of ADDLs contain oligomers of a wide range of sizes and are OC-positive, suggesting that ADDLs contain fibril-like structures. Studies from NMR, EPR, and cryo-EM have shown that the vast majority of fibril structures are parallel in-register β-sheets.^3,48,57,58^ Therefore, these results also support the parallel in-register structure in ADDLs.

FTIR studies^30^ of ADDLs show an amide I peak at approximately 1695 cm^–1^, which has been interpreted as a fingerprint of antiparallel β-sheets. This FTIR signature peak has also been found in various other Aβ oligomer preparations.^59−65^ It is worth noting that the amide I peak at ∼1695 cm^–1^ was established as a signature of antiparallel β-sheets using model proteins and peptides.^66−68^ It remains to be seen whether this peak also represents a unique identifier of antiparallel structures in structurally heterogeneous Aβ oligomers. The difference in structural interpretations between FTIR and other techniques remains to be solved.

## Conclusions

We obtained structural information on Aβ42 ADDL oligomers using site-directed spin labeling at 37 unique residue positions. The EPR data revealed a loosely packed N-terminal region at residues 1–14. The C-terminal residues 15–22 and 30–40 displayed low spin label mobility, consistent with a β-turn-β structural motif. The single-line EPR feature at multiple residue positions indicates a parallel in-register β-sheet structure, with residues 16–21 and 34–38 forming the structural core. In agreement with these findings, recent NMR studies^53,54^ also show parallel β-sheet structures in Aβ42 oligomers prepared with similar protocols. Collectively, these findings suggest that fibril-like parallel β-sheet structures may be a common structural class for Aβ oligomers.

## Methods

### Preparation of Aβ42 Proteins and Spin Labeling

Recombinant Aβ42 proteins were expressed and purified as a fusion protein of GroES-ubiquitin-Aβ42 and GroES-ubiquitin was then cleaved off using the deubiquitylating enzyme Usp2-cc.^69,70^ Single cysteine mutants were introduced using site-directed mutagenesis as previously described.^39^ Detailed expression and purification procedures have been previously described.^41,45^ For spin labeling, the spin labeling reagent MTSSL (1-oxyl-2,2,5,5-tetramethylpyrroline-3-methylmethanethiosulfonate, AdipoGen Life Sciences) was used to attach the commonly used spin label R1. Detailed labeling protocols have been previously described.^39,41,43^ Spin labeling efficiency was assessed with mass spectrometry, and only samples with >95% labeling efficiency were used in subsequent studies. All spin-labeled Aβ42 proteins were lyophilized and stored at −80 °C.

### Preparation of Aβ42 ADDL Oligomers

Lyophilized Aβ42 proteins in powder form were dissolved in cold hexafluoroisopropanol (HFIP) to 100 μM Aβ42 concentration and incubated at room temperature for 24 h with shaking at 1000 rpm. The HFIP was evaporated in a chemical hood overnight, leaving a film of the Aβ42 proteins. To prepare Aβ42 ADDL oligomers, the HFIP-treated Aβ42 was first dissolved in anhydrous DMSO at 5 mM and then diluted with ice-cold phenol red-free F12 medium to a 100 μM Aβ42 concentration with brief vortexing. Next, the solution was incubated in the refrigerator (4 °C) for 24 h. The sample volume at this step was typically 500 to 1000 μL. Then, the sample was centrifuged at 14,000*g* for 10 min at 4 °C to separate the pellet containing insoluble aggregates from the supernatant containing ADDLs. For TEM studies, ADDL oligomers were used directly. For EPR studies, the ADDLs were concentrated using a 30-kDa ultrafiltration filter to a final volume of ∼15 μL, immediately followed by EPR measurements. One oligomer sample was prepared with each spin-labeled Aβ42 variant, except for S8R1 and F19R1, for which two oligomer samples were prepared.

### Transmission Electron Microscopy

For transmission electron microscopy, 5 μL of Aβ42 ADDL oligomers were applied on glow-discharged copper grids (400 mesh Formvar/carbon film, Ted Pella) and stained with 2% uranyl acetate. The grids were examined by using a FEI T12 electron microscope with an accelerating voltage of 120 kV.

### EPR Spectroscopy and Spectral Simulations

For EPR measurements, spin-labeled Aβ42 ADDL oligomers were loaded into glass capillaries (VitroCom) with the capillaries sealed at one end. EPR spectra were collected at the X-band using a Bruker EMXnano spectrometer at room temperature. Modulation amplitude was optimized for the individual spectrum (typically 2 G). Typically, 100 to 400 scans were averaged for each EPR spectrum with a sweep time of 10 s. To quantitatively analyze the EPR spectra, spectral simulations were performed using the program MultiComponent, written by Dr. Christian Altenbach at the University of California Los Angeles. A microscopic order macroscopic disorder model was used to describe the motion of spin label.^71^ A least-squares fit of the user-defined spectral parameters was performed by using the Levenberg–Marquardt algorithm. Detailed fitting procedure has been previously described.^41^ For all the fits, the magnetic tensor *A* and *g* were set as *A*_*xx*_ = 6.2, *A*_*yy*_ = 5.9, *A*_*zz*_ = 37.0, and *g*_*xx*_ = 2.0078, *g*_*yy*_ = 2.0058, *g*_*zz*_ = 2.0023 as described previously.^50^ For the structured component, an anisotropic model of motion was used for R1 by including an order parameter (*S*). For anisotropic simulations, the diffusion tilt angles were fixed to (α,β,γ) = (0,36°,0) for *z*-axis anisotropy as previously described.^50^ For the disordered component, an isotropic model was used for R1. The number of fitted parameters was kept at a minimum. We found that satisfactory fits were obtained with three fitted parameters: rotational diffusion constant (*R*) and order parameter (*S*) to describe the motion of the spin label and Heisenberg exchange frequency (ω) to represent the rate of spin exchange. Rotational correlation time (τ) was calculated by using τ = 1/(6*R*). The fitting procedure was allowed to converge without intervention to obtain the fitting parameters.

## Abbreviations

ADDL: Aβ derived diffusible ligand
DMSO: dimethyl sulfoxide
PBS: phosphate-buffered saline
SDS: sodium dodecyl sulfate
DPC: dodecyl phosphocholine
FTIR: Fourier transform infrared spectroscopy
AFM: atomic force microscopy
SEC: size exclusion chromatography
EPR: electron paramagnetic resonance
NMR: nuclear magnetic resonance
TEM: transmission electron microscopy
WT: wild-type
HFIP: hexafluoroisopropanol
